# Low-dose oral immunotherapy in immunoglobulin E-mediated food allergies

**DOI:** 10.3389/fimmu.2024.1321863

**Published:** 2024-02-01

**Authors:** Dongxia Ma, Rongfei Zhu

**Affiliations:** ^1^ Department of Allergy, Tongji Hospital, Tongji Medical College, Huazhong University of Science and Technology, Wuhan, China; ^2^ Institute of Allergy and Clinical Immunology, Tongji Hospital, Tongji Medical College, Huazhong University of Science and Technology, Wuhan, China

**Keywords:** low-dose OIT, conventional OIT, mechanisms, hypoallergenic foods, efficacy and safety

## Abstract

Nowadays, the management of food allergies has increasingly moved from conventional oral immunotherapy (OIT) to low-dose OIT or low-dose OIT utilizing hypoallergenic foods. This shift is largely because the latter appears to induce oral tolerance with fewer adverse effects than the former. However, the mechanisms underpinning such differences remain unclear. To better understand these mechanisms, we conducted a comparative study scrutinizing the mechanisms of OIT, especially those of low-dose desensitization. We also summarized articles on low-dose OIT and low-dose OIT using hypoallergenic foods. We examined the efficacy, safety, and immunological parameters of low-dose OIT and those of low-dose OIT with hypoallergenic foods with the aim of shedding some light on low-dose OIT and its therapeutic application in inducing oral tolerance for individuals with food allergies.

## 1 Introduction

Food allergy, the prevalence of which is on the rise in both developed and developing countries, adversely impacts individuals’ quality of life and wellbeing (1). At times, it may trigger severe and even life-threatening allergic reactions, such as anaphylactic shock, which is induced by immunoglobulin E (IgE)-mediated immediate hypersensitivity reactions (2). Presently, there are primarily two strategies for managing IgE-mediated food allergies: a strict elimination diet devoid of the offending foods and allergen immunotherapy. The former is currently the most common approach worldwide, but it may lead to malnutrition (3) Additionally, it does not eliminate the risk of allergic reactions due to unintentional consumption of the allergenic food. Consequently, the latter is gaining more traction in food allergy treatment.

Food allergen immunotherapy is categorized into several types based on the administration route, such as oral immunotherapy (OIT), sublingual immunotherapy (SLIT), and epicutaneous immunotherapy (EPIT). Among these, OIT is more commonly employed than the others (4). However, its clinical use is sometimes constrained due to its potential to trigger severe allergic reactions. To mitigate or even prevent allergic reactions during OIT, numerous attempts have been made, including using low-dose OIT and low-dose OIT combined with hypoallergenic foods for severe food allergic patients. The term “low-dose” in some clinical trials often refers to a lower maintenance dose and target dose than that of conventional OIT (typically ranging from 5% to 40%), although a clearer definition for low-dose OIT is yet to be established. Studies have demonstrated that low-dose OIT exhibits similar effectiveness and a better safety profile compared to conventional OIT (5, 6). Our study aimed to delve into the mechanisms underlying such a difference by reviewing and comparing the efficacy and safety of these approaches to food allergy, with a focus on low-dose OIT and low-dose OIT with hypoallergenic foods.

## 2 Oral immunotherapy and low-dose oral immunotherapy

Oral immunotherapy (OIT) has been employed in the treatment of IgE-mediated food allergies to induce desensitization and tolerance. Offending food allergens are introduced in escalating doses until the established maintenance dose is reached. OIT typically includes an initial dosage escalation phase, also referred to as the initiation phase, followed by a maintenance phase with a slightly higher dose. An oral food challenge (OFC) is then performed to assess the therapeutic effect. Some individuals will enter a non-response period, also known as the sustained unresponsiveness state (7). However, studies have shown that this clinical tolerance gradually diminishes once regular and consistent intake of the offending food is withdrawn (8, 9). In other words, OIT may offer temporary protection after a period of maintenance dose therapy. To maintain a sustained desensitization effect, many programs recommend indefinite dosing at some frequency even after the patients demonstrate sustained unresponsiveness (8–10).

Low-dose OIT involves a lower maintenance dose and target dose than conventional OIT (11). Previous clinical studies on conventional OIT showed that the maintenance dose is usually 5,000-6,000 mg of wheat protein (12–15), 4,950- 6,600 mg (150-200ml cow’s milk) milk protein (16–20), 3,300-13,600 mg of egg white protein (21–24), 2,000-4,000 mg of peanut protein (10, 25–27), and 1,200 mg walnut protein (28). The selection of maintenance doses in these studies appears arbitrary, spanning a broad range, or based on expected accidental exposures, which may not be optimal. Nevertheless, these studies indicate that OIT with such maintenance doses can enhance the tolerance threshold for allergenic food. Our review included low-dose OIT studies in which the maximum maintenance dose is 1,445 mg for wheat protein, 850 mg for cow’s milk protein, 1,550 mg for egg protein, 300 mg for peanut protein, and 75 mg for walnut protein.

It has been shown that low-dose OIT can protect against reactions from accidental exposure to culprit food and reduce the occurrence of treatment-related allergic reactions during OIT (6, 29–31). The incidence of allergic reactions is rather low in low-dose OIT. Moreover, moderate to severe treatment-related symptoms are rare (6, 32, 33). Naturally, low-dose OIT has been used for the treatment of patients severely allergic to milk, egg, wheat, peanut, and walnut with a slower dose escalation phase (5, 32, 34–36).

Considering the potential risks in OIT, to minimize anaphylaxis for subjects with severe food allergies, several studies have been conducted using a combination of low-dose OIT with hypoallergenic foods (37–39), such as baked milk, heated milk, and hydrolyzed eggs. While the definition for hypoallergenic foods remains contentious, a few OIT studies with hypoallergenic foods have been conducted. The reason for decreased allergenicity is that the heating process induces food denaturation through conformational changes of the protein epitope (37). These foods have low allergenicity, but they can all induce oral tolerance (38, 40, 41). However, data show that OIT with hypoallergenic food desensitizes to a lower eliciting dose and desensitizes fewer patients than the conventional dose (37, 42).

## 3 Mechanisms of oral immunotherapy and low-dose oral immunotherapy

The immunologic mechanisms underlying oral tolerance induced by OIT are extraordinarily complex. Both innate and adaptive immunity play a role in this process. For instance, it has been demonstrated that OIT influences not only adaptive but also innate immunity in children with cow’s milk allergy mediated by specific IgE (sIgE) (40). Typically, food OIT induces tolerance by modifying the immune response. Overall, the mechanisms by which low-dose and conventional OIT induce immune tolerance are similar, yet there are still some differences.

### 3.1 Dendritic cells

Dendritic cells (DCs) are well recognized for their crucial role in modulating innate immunity through the toll-like receptors (TLRs) signaling pathway. As major antigen-presenting cells, DCs facilitate Th2 differentiation during allergy development, thereby acting as a bridge between innate and adaptive immunity. They play a pivotal role in tolerance induction during conventional OIT. OIT has been found to increase IL-10 production in myeloid DCs, which helps inhibit FcϵRI-dependent pro-inflammatory responses. Some studies, considering the FcϵRI-dependent pathway, have suggested that changes in DCs’ immune response during OIT were related to allergen-specific IgE and IgG (43). Interestingly, OIT may enhance IFN-α secretion and downregulate IL-6 production from plasmacytoid DCs stimulated with TLR7 and/or TLR7/9 agonists, leading to reduced IL-13 release (43).

Concurrently, a clinical trial demonstrated that peanut OIT can decrease inflammatory cytokine production from DCs through TLR expression modulation, promoting the differentiation of regulatory T cells and inhibiting Th2 formation (44). It has also been shown that peanut OIT suppresses Th2 inflammatory responses by reducing the expression of CD40, human leukocyte antigen DR, and even CD86, but increasing CD80 expression on DCs (45).

DCs also play a significant role in tolerance induction during low-dose OIT. A study on peanut low-dose OIT in mice indicated that low-dose OIT induces the generation of CD103^+^DCs. These DCs belong to a subset of regulatory DCs and can positively regulate the formation of regulatory T cells by decreasing Foxp3 methylation, which is related to the suppressive function of Treg cells (43, 46).

### 3.2 T cell response

T cell response is involved in both conventional OIT and low-dose OIT. Antigen-specific CD4^+^Th2 cells significantly decrease as the duration of OIT extends. Antigen-specific Th2A cells (CD4^+^CD45RO^+^CD27-CD45RBloCRTH2^+^CD49d^+^CD161^+^) undergo a sharp decrease, especially in the first three months of OIT, followed by a gradual decline. The potential mechanisms behind this cell reduction remain unclear, although deletion, anergy, and exhaustion may all contribute (44). There was a notable decrease in the frequencies of IL-4^+^, IL-9^+^, and IL-10^+^ peanut-reactive CD4^+^T cells among total CD4^+^T cells in peanut-allergic individuals following OIT (47). Additionally, it was discovered that, compared with desensitization, higher frequencies of both IL-4^+^CD4^+^T cells and IFN-γ^+^ CD4^+^T cells post-OIT displayed a significant negative correlation with sustained unresponsiveness (47). Meanwhile, there was a significant reduction in the Th2-polarization surrogate marker OX40 expression in CD4^+^T cells in peanut-allergic individuals after desensitization (47). The study by Michael Kulis et al. (48) demonstrated a significant increase in peanut-responsive CD4^+^T cells during the first four and eight months of peanut OIT in both high- and low-dose groups compared to baseline. However, this increase in peanut-responsive CD4^+^T cells was transient and was not sustained beyond eight months of OIT. Furthermore, no significant differences were detected between both high- and low-dose groups. Abhinav Kaushik et al. (47) demonstrated that in peanut-allergic individuals, not only were lower frequencies of naive CD8^+^T cells observed, but also terminally differentiated under long term antigenic stimulation CD57^+^CD8^+^T cell subsets at baseline, which were reported to be highly proinflammatory correlated with sustained unresponsiveness after OIT. Furthermore, the frequency of naive CD8^+^T cells was significantly and positively associated with peanut-specific and Ara h2-specific IgE levels before OIT.

It has been demonstrated that Treg cells are important in inducing food allergen tolerance during OIT (44). This includes Foxp3^+^Treg, latency-associated peptide (LAP)^+^Treg, and Type 1 regulatory T (Tr1) cells (49). These Tregs are typically induced during OIT. Foxp3+Treg represents a classic regulatory T cell, with the expression of the transcription factor Foxp3 being essential for its functionality. The reduced methylation of the Foxp3 locus plays a significant role in sustaining prolonged clinical desensitization in patients who have achieved clinical tolerance following peanut OIT (45). It has been demonstrated that LAP^+^ Treg is associated with oral tolerance (50), and it may induce Foxp3^+^ Treg differentiation, among which the key factor is TGF-β concentration. Tr1 cells are characterized by high expression of IL-10, and their hallmark feature is co-expression of CD49b and lymphocyte activation gene 3 (LAG3). Tr1 cells promote oral tolerance mainly through IL-10 and IL-21 production. Additionally, regulatory CD8^+^T cells and γδ^+^ T cells have assisted in oral tolerance, although they may not play an essential role in orally induced tolerance (49). A high- and low-dose peanut OIT study in which 3,000 mg and 300 mg peanut protein were set as the daily maintenance dose respectively suggested that Tregs increased transiently in the fourth month. However, the increasing trend was not sustained throughout the course of therapy. Moreover, in terms of Treg numbers, no significant differences were detected between high-and low-dose groups (48).

Currently, while there are numerous studies focusing on T-cell responses in conventional OIT, there are fewer studies addressing low-dose OIT. Consequently, to discern the differences in T-cell responses between conventional and low-dose OIT, additional research is necessary.

### 3.3 Basophil response and mast cells

Mast cells and basophils serve as the principal immune effector cells in the IgE-mediated food allergy. The changes they undergo during OIT are closely related to the development of tolerance.

#### 3.3.1 Basophil response

It is well established that OIT leads to a reduction in basophil activation (51, 52). Basophil activation tests (BATs) are conducted *in vitro* using whole blood to assess degranulation in response to allergen stimulation. The expression of CD63 and CD203c are typically measured by flow cytometry to evaluate basophil activation. CD63 is expressed on granules and fuses with the cell membrane upon degranulation. CD203c, constitutively expressed on the cell membrane, is upregulated upon activation. OIT significantly decreases the levels of CD63 and CD203c on the cell membrane (53).

The suppression of basophil activation is a crucial component of OIT-induced immune tolerance during conventional OIT. This suppression can occur as early as the first few months after initiating OIT (54). Lower basophil reactivity is observed throughout the OIT maintenance phase, playing a vital role in inducing tolerance. It has been demonstrated that peanut OIT suppresses basophil activation. Furthermore, the assessment of peanut-specific basophil activation is a useful predictor for the outcome of OIT and can even differentiate between transient desensitization versus sustained unresponsiveness (SU) after concluding OIT (55). However, basophil reactivity often slowly rebounds once OIT is discontinued, with basophil reactivity dramatically reversing at 4 to 6 weeks after cessation of treatment in subjects receiving peanut OIT (56). Additionally, there is a smaller increase in basophil reactivity in those achieving tolerance compared to those who have not. Therefore, reducing basophil reactivity is essential for achieving clinical desensitization and tolerance (44).

The reduction in basophil activation during the initiation phase is independent of serum IgE (sIgE) levels, which tend to trend upward during the initial dosage escalation phase. Instead, it may be correlated with the escalating antigen dose that contributes to basophil anergy. Interestingly, it has been demonstrated that basophil anergy induced by OIT is non-specific (57). As OIT progresses, the decrease in basophil activation is partly due to reduced IgE. Moreover, basophil activation is suppressed by IgG4, induced by OIT, through its activity on FcγRIIb, the only inhibitory IgG receptor, which activates phosphatases inhibiting FcϵRI signaling (58). Depletion of IgG in plasma abrogates the suppression of basophil activation *in vitro* (59). Additionally, a decrease in basophil activation capacity often indicates desensitization or tolerance. Furthermore, IgA, which is upregulated in response to OIT, especially in the intestine, plays a significant inhibitory role in basophil activation (59). Importantly, Tregs, as crucial immunoregulatory cells, also contribute to the suppression of basophils (58).

The suppression of basophil activation is also significant in food tolerance induced during low-dose OIT. It has been demonstrated that the suppression of basophil activation induced by OIT can last for a certain period, which can sustain for 4 weeks without peanut dosing both in subjects receiving high- and low-dose peanut OIT (48). Additionally, high- and low-dose peanut OIT have almost identical effects on basophil suppression (48). Notably, in participants undergoing hypoallergenic foods OIT with dehydrated egg white (EW) powder, both CD203c^+^ and CD63^+^ basophils undergo a more significant decrease over time than those in the placebo group (51).

#### 3.3.2 Mast cells

In the context of OIT, while basophil activation decreases, mast cell activity also exhibits a similar downward trend, albeit their roles in OIT are not entirely identical. Despite this, the high-affinity IgE receptor, FcϵRI, retains its ability to transduce signals when bound to serum IgE (sIgE) in desensitized mast cells. A noticeable decrease in mast cell degranulation is observed, the underlying mechanism of which involves the restriction of phosphorylation and dephosphorylation of cofilin in response to antigen challenge in desensitized mast cells. As a result, cofilin-mediated actin turnover is inhibited, leading to the stability of F-actin filaments in desensitized mast cells. This further limits calcium flux, which is crucial for mast cell degranulation induced by FcϵRI-mediated signaling, as demonstrated in mouse models of food OIT (54, 60). However, the desensitization of bone marrow-derived mast cells *in vitro* is associated with the formation and internalization of small IgE-FcϵRI clusters (61). Furthermore, mast cell desensitization partially depends on the internalization of antigen-specific IgE on the mast cell surface in both *in vivo* and *in vitro* mouse models. Exposing mast cells to progressively increasing amounts of antigen rapidly can both enhance the internalization of antigen-specific IgE on the mast cell surface and desensitize these cells in an antigen-specific manner (62). Allergen-specific IgG, which inhibits mast cell activation induced by sIgE through steric blockade of antigenic epitopes and signaling via the inhibitory Fc receptor FcγRIIb, has garnered considerable interest (63). Simultaneously, antigen-specific IgA, the most abundant antibody isotype in the lower digestive tract, also mitigates mast cell activation instigated by sIgE through its binding to mast cells, this process is dependent on calcium and sialic acid. It also inhibits the phosphorylation of Syk, a key proximal protein kinase in FcϵRI signaling, and suppresses cytokine expression in mast cells (63). However, there is currently no data to suggest differences in mast cell activation between conventional and low-dose OIT.

### 3.4 Cytokine response

Various types of cytokines are implicated in both conventional OIT and low-dose OIT. Th2 inflammatory cytokines, which mediate food allergies, are reduced by OIT, notably IL-5, IL-4, and IL-13. Peanut OIT significantly diminished type 2 cytokines IL-4, IL-5, IL-9, and IL-13 at week 104 in supernatants of peanut-stimulated peripheral blood mononuclear cells (PBMCs) (47). Meanwhile, Katharina Blumchen et al. (31) demonstrated that among peanut-allergic children receiving low-dose peanut OIT, there was a significant reduction in IL-2, IL-4, and IL-5 production by PBMCs after 16 months of low-dose OIT compared to the placebo group. Moreover, a study of high- and low-dose oral immunotherapy by Michael Kulis et al. suggested that Th2- and Th9-type cytokines (IL-5 and IL-13 for Th2-type inflammation and IL-9 for Th9-type inflammation) all decreased throughout the course of OIT in both groups on high- and low-dose OIT conducted for peanut-allergic children with 300 mg or 3,000 mg peanut protein, respectively (48). There were no noticeable differences in these cytokines’ changes between the high- and low-dose OIT groups.

What is worth highlighting is that the trend of IL-10, IFN-γ, and TNF-α changes varies in different OIT trials (44). It was shown that a modest increase of Th1 cytokine and IL-10 was conducive to tolerance formation in a mice food OIT model. Yet, Katharina Blumchen et al. (31) reported that in peanut-allergic children receiving low-dose peanut OIT, there was a significant reduction in IL-10 induced by peanut stimulation of PBMCs after 16 months of low-dose OIT compared to the placebo group. Surprisingly, IL-10, TNF-α, and IFN-γ in sera all decreased in any subject receiving either 300 mg or 3,000 mg of peanut OIT. As for the Th2- and Th9-type cytokines, no evident differences between the high- and low-dose OIT groups were detected with regard to IL-10, TNF-α, and IFN-γ production. Moreover, IL-17 significantly declined in both high- and low-dose OIT, although the difference between both groups was not significant (48). Therefore, the role that these cytokines play in inducing tolerance necessitates further study.

### 3.5 Humoral immune response

Humoral immune responses to OIT primarily encompass specific IgE (sIgE) and specific immunoglobulin G (sIgG) changes at various stages of OIT. Both are crucial in conventional OIT. At the onset of OIT, the levels of both food-sIgE and food-specific component IgE escalate, and they gradually decrease with the progression of OIT. It was demonstrated that a lower baseline level of food-sIgE correlates with desensitization or tolerance. Concurrently, food-specific component IgEs, such as ovalbumin and casein sIgE, have been proven to be reliable predictors of desensitization or tolerance (38, 39). However, Kiyotake Ogura et al. (6) revealed that there was no significant connection between sustained unresponsiveness (SU) and the degree of food-sIgE reduction.

The baseline level of food-specific IgG4 indicates allergen exposure. If it is relatively high before OIT and the patients do not exhibit severe allergic symptoms once the culprit food is consumed, this usually indicates less severe allergic reactions at baseline (44). Serum food-sIgG increases gradually with OIT, and these trends have been widely reported in milk, egg, peanut, and wheat OIT (32, 34, 36, 37, 44, 64). Moreover, plasma from subjects following peanut OIT inhibits basophil activation *in vitro*, and the inhibiting effect is abrogated after IgG depletion (59, 65). Although all subclasses of food-specific IgG (IgG1, IgG2, IgG3, and IgG4) increased during OIT, IgG4 accounted for most of the inhibiting effects (53). Additionally, there is a significant increase of IgG4 in patients receiving OIT with SU (58). Allergen components research shows that all IgG4 to α-lactalbumin, β-lactoglobulin, and casein increase during milk OIT, and both salivary and serum food-specific IgG4 may be useful as predictors of OIT outcome (53).

As with conventional OIT, sIgE and sIgG4 also have a significant effect on low-dose OIT. There was a dramatic increase of sIgG4 after slow low-dose OIT in many clinical trials (32, 34, 37, 66). Additionally, increased peanut-IgG4/IgE ratios were detected in both high- and low-dose peanut oral immunotherapy in peanut-allergic children (48). A low-dose OIT with low-egg-allergen cookies trial showed that the ratios of ovomucoid (OM)-sIgG4/OM-sIgE after OIT were significantly higher compared to baseline (41). Meanwhile, a randomized trial of low-dose oral immunotherapy for pediatric cow’s milk-induced anaphylaxis with heated and unheated milk indicated that casein-sIgG4 significantly increased in both groups from baseline. However, a significant increase of β-lactoglobulin-sIgG4 was detected only in the unheated milk group (37). The reason may be that β-lactoglobulin conformational changes and epitopes that elicit IgG4 production are destroyed during the heating process. In a peanut low-dose OIT model in mice, IgG1and IgG2a in serum significantly increased. Considering that mice do not have an exact equivalent of the human IgG4 subclass, we can speculate that the increase in IgG1 and IgG2a contributes to the formation of immune tolerance (46).

IgG and IgG4 levels might not be the only deciding factor in OIT-induced immune tolerance. It has been demonstrated that there are similar levels of Ara h2-sIgG and IgG4 in individuals receiving peanut OIT with sustained or transient non-responsiveness, and Ara h2 mAbs from sustained and transient non-responders have similarly high affinity. However, there are some differences in antibody responses against conformational epitopes of the immunodominant allergen Ara h2 between individuals with sustained and transient non-responsiveness. There are three conformational epitope bins for Ara h2 designated as bins 1, 2, and 3. Furthermore, these three bin 3 antibodies are the rarest ones, and they have only been identified in sustained non-responders. Additionally, they may more effectively disrupt allergen-IgE interactions and suppress basophil degranulation. Therefore, the unique Ara h2-specific neutralizing antibodies were extremely important in promoting the durability of allergic tolerance (67).

OIT may also alter the binding of sIgE and sIgG4 to culprit food peptides. In children receiving cow’s milk OIT, their sIgEs bound to cow’s milk peptides significantly decreased while IgG4 binding increased (68). Moreover, OIT decreased both the affinity and quantities of epitope-specific IgE antibodies. In recent years, it has been demonstrated that, in peanut-allergic individuals, OIT-induced IgG and IgE exhibit a high degree of overlap in their specificity towards antigens. Additionally, IgG and IgE exhibit strikingly similar antibody footprints, suggesting that they share related clonal lineages or convergent evolution of specific IgE and IgG B cells (69).

It has been suggested that IgA expression increased over the course of OIT, including IgA1 and IgA2 specific for allergen and allergen components. In particular, IgA2 level rose dramatically in OIT treatment responders that was more stable than IgA1. And IgA2 was mainly distributed in mucosal tissue, which may reflect a local IgA response during OIT in gastrointestinal mucosa (58). Furthermore, in the study by Akihiro Maeta (41), it was demonstrated that serum OVA-sIgA2 levels significantly increased compared to baseline in the severe egg-allergic children receiving low-dose oral immunotherapy with low-egg-allergen cookies. Moreover, the ratios of OM-sIgA2/OM-sIgE after OIT were significantly higher than before OIT. In a model of peanut low-dose OIT in mice, IgA significantly increased (46). It has been discovered that there was a significant increase of IgA and IgA2 to egg white (EW) in individuals that achieve unresponsiveness to egg. Thus, IgA may be a potential reliable predictor of desensitization or tolerance (53).

## 4 Summary of low-dose OIT trials

**Table 1** provides an overview of studies focusing on low-dose OIT. The outcomes of these OIT trials can vary substantially due to the diverse measures employed in the studies. These measures include factors such as the increment in dose during the initial dosage escalation phase, the maintenance dose, the duration of the maintenance phase, and doses administered post-maintenance. As a result of these varying protocols, differences can be observed in terms of efficacy, safety outcomes, and immunological parameters.

**Table 1:** Studies on low-dose oral immunotherapy.

Food allergen	Reference	Design	Sample size and age	Patient characteristics	Maintenance dose	OIT duration	Efficacy outcome	Safety outcome	Immunological parameter
**Wheat**	Nowak Wegrzyn et al. (2019/2) (32)	Multicenter, double blind, randomized, placebo-controlled trial	Low dose OIT: n = 23, 6.8–11.3 years; Placebo: n = 23, 5.4–10.8 years	Positive OFC with <1,443 mg of wheat protein	OIT group: 1,445 mg of wheat protein Placebo group: completely eliminated wheat intake	1 year	Desensitization to 4,443 mg wheat protein OIT: 52.2% Placebo: 0% (P < 0.0001)	Any AE per dose: 15.4%; severe AE: 0.04%.	Wheat- and ω-5G–sIgE levels did not differ; the low-dose OIT median sIgG4 level was greater than placebo (wheat, P <0.0005; ω-5G, P<0.0001)
	Nagakura et al. (2020/5) (34)	Open-label, non-randomized, historically controlled trial	Low dose OIT: n = 16, 5.8–10.7 years; Historical control: n = 11, 5.0–8.1 years	Positive OFC with 53 mg of wheat protein and a history of anaphylaxis	OIT group: 53 mg of wheat protein Placebo group: completely eliminated wheat intake	1 year	StU to 400 mg OIT: 25% Control: 0.0% (P = 0.07)	Any AE per dose: in the hospital: 32.1%; at home: 4.1%.	Wheat-and ω-5G-sIgE levels significantly decreased at 1 year; wheat- and ω-5G-sIgG and sIgG4 levels significantly increased at 1 month.
	Ogura et al. (2020/9) (6)	Multicenter, open label, randomized, non-placebo- controlled study	Low dose OIT: n = 12, 4.5–5.8 years; High dose OIT: n = 12, 3.7–5.5 years	Positive OFC with 78–650 mg of wheat protein	Low dose: 650 mg of wheat protein; high dose: 2,600 mg of wheat protein	1 year	StU to 2,600 mg, Low dose OIT: 16.7% High dose OIT: 50% (*P* = 0.193)	Any AE per dose: low dose OIT: 4.76%; high dose OIT: 8.82% (p<0.05)	Wheat-sIgE significantly decreased in both groups (p<0.05)
	Shiro Sugiura et al. (2020/10) (33)	Open label non-randomized controlled trial	Low dose OIT: n = 35, 4–6 years; Control: n = 10, 5–6.8 years	Positive OFC with 226 mg of wheat protein	Low dose: > 10 times than the maximal tolerated dose at baseline Control: without wheat protein.	1 year	Desensitization to 226 mg OIT: 37.5% Control: 10% (P = 0.13)	Any AE per dose: 0.64%	Wheat-and ω-5G-sIgE significantly decreased (wheat, P =0.004; ω-5G, P=0.02);
**Milk**	Noriyuki Yanagida et al. (2015/9) (35)	Single-center, case-control study	Low dose OIT: n=12, 6.7-12.8 years; Control: n=25, 5.8-9.7years	Positive OFC with 3 ml of CM	Low dose OIT: 3 ml of CM; control group: completely eliminated their milk intake	1 year	Unresponsive to 3 ml of milk OIT: 58.3% Control: 13.8% (P = 0.018)	Any AE per dose: in the hospital, 57.1%; at home, 19.5%.	Low dose OIT: Casein-sIgE significantly decreased; the β-lactoglobulin sIgE levels did not differ; control group: milk-sIgE levels did not differ.
	Ogura et al. (2020/9) (6)	Multicenter, open label, randomized, non-placebo- controlled study	Low dose OIT: n = 13, 6.1–10.4 years; High dose OIT: n = 13, 6.0–7.7 years	Positive OFC with 102–850 mg of CM protein	850 mg of CM protein High dose: 3,400 mg of CM protein	1 year	StU to 3,400 mg Low dose OIT: 15.4% High-dose OIT: 7.7% (P > 0.999)	Any AE per dose low dose OIT: 10.3% high-dose OIT: 15.4% (P < 0.05)	CM-sIgE and casein-sIgE significantly decreased (p<0.05)
	Shiro Sugiura et al. (2020/10) (33)	Open label non- randomized controlled trial	Low dose OIT: n = 41, 4–7 years; Control: n = 9, 4–6 years	Positive OFC with 287 mg of CM protein	Low dose > 10 times than the maximal tolerated dose at baseline Control: without milk protein.	1 year	Desensitization to 287 mg of CM protein OIT: 37.5% Control: 0.00% (P = 0.042)	Any AE per dose: 0.47%.	CM-sIgE and Casein -sIgE significantly decreased in OIT group (p were both <0.001)
**Egg**	Noriyuki Yanagida et al. (2017/1) (36)	Single-center, randomized, placebo-controlled, low dose trial	Low dose OIT group: n=21, 6.2-18.7 years; Placebo group, n=12, 5.9-12 years	Past histories of an anaphylactic reaction or high EW-sIgE >30 kU/L	Low dose OIT group, 194 mg of egg protein; placebo group, complete egg avoidance	12 months	STU to 194 mg egg protein OIT: 71% Control: 0%; STU to 1/2 of a whole egg (3,104 mg egg protein) OIT: 33% Control:0%	Any AE per dose: in hospital 58.8%; at home: 6.5%; No severe symptoms occurred.	Only in the OIT group, EW-sIgE and ovomucoid-sIgE significantly decreased; EW-sIgG, ovomucoid-sIgG, EW-sIgG4, and ovomucoid-sIgG4 significantly increased.
	Ogura et al. (2020/9) (6)	Multicenter, open label, randomized, non-placebo- controlled study	Low dose OIT: n = 25, 5.2–8.9 years; High dose OIT: n = 26, 4.7–9.2 years	Positive OFC with 194–1,550 mg of whole egg protein	Low dose group: 1,550 mg egg protein; High dose group: 6,200 mg egg protein	1 year	StU to 6,200 mg egg protein, Low dose OIT group: 20%; High-dose OIT group: 26.9% (P = 0.743)	Any AE per dose: low dose OIT: 8.74%; high dose OIT: 10.9% (P < 0.05)	EW-sIgE and OM-sIgE significantly decreased in both groups (p<0.05)
	Shiro Sugiura et al. (2020/10) (33)	Open label non- randomized controlled trial	Low dose OIT: n = 104, 5–7 years; Control: n = 29, 5–8 years	Positive OFC with 983 mg of hen’s egg protein	Low dose OIT: > 10 times than the maximal tolerated dose at baseline; control: without egg protein.	1 year	Desensitization to 983 mg, OIT: 34.7% Control:11.1% (P = 0.018)	Low dose OIT group, any AE per dose: 0.60%.	EW-sIgE and OM-sIgE significantly decreased in the low dose OIT group (p were both <0.001).
**Peanut**	Brian P. Vickery et al. (2017/1) (5)	Single-center case-control, randomized, double-blinded study	9 to 36 months n=40; Low dose OIT: 20; High dose OIT:20.	Reacted to peanut during an entry food challenge	Low dose OIT: 300 mg peanut protein; high dose OIT: 3,000 mg peanut protein	29 months	StU to 5g peanut protein, 300 mg arm: 85% 3,000 mg arm: 71%	Any AE per dose high dose OIT:1.1%; low dose OIT: 0.6% None severe.	Peanut-sIgE significantly decreased (p<0.0001). There was no significant difference between both groups in sIgE changes.
	Kenichi Nagakura et al. (2018/8) (70)	Open label non- randomized controlled trial	5 to 18 years, low dose OIT: n =24; historical control group: n=10	With a history of anaphylaxis or high levels of peanut-sIgE (>50 KUA/L)	Low dose OIT: 133 mg peanut protein; control group: without peanut protein.	1 year	StU to 795mg peanut protein, low dose OIT: 33.3% Control: 0%	Low dose OIT: any AE per dose: in hospital 66.4%; at home: 7.4%; No severe symptoms occurred.	Only in the OIT group: peanut and Ara h2–sIgE levels dramatically increased after 1 month, and decreased at 3, 6, and 12 months. Peanut- and Arah2-sIgG and sIgG4 significantly increased at 1 month.
	Katharina Blumchen et al. (2019/2) (31)	Multicenter, randomized, placebo-controlled, low dose trial	3-17 years old, low dose OIT: n=31; placebo group, n=31	Positive OFC with 4,500 mg peanut protein	Low dose OIT group, 125-250 mg peanut protein; placebo group: 125-250 mg placebo	16 months	StU to ≥300 mg peanut protein, OIT:74.2%; placebo: 16.1%. StU to≥4,500 mg peanut protein: OIT 41.9%. placebo 3.2%;	Peanut-OIT 4.3%; placebo-OIT 1.2%;	Compared with the placebo group, peanut-specific IL-4, IL-5, IL-10, and IL-2 production by PBMCs significantly decreased, and peanut sIgG4 significantly increased in the peanut-OIT group.
**Walnut**	Koki Sasamoto et al. (2021/7) (71)	Case reports	Over 5 years old, low dose OIT: n=3	Positive for OFC with 75 mg walnut protein	Low dose OIT: 75 mg walnut protein	1 year	All three patients were StU to 450 mg peanut protein after 12, 14, and 24 months, respectively.	Any AE per dose: 3.2%	Walnut-sIgE level and Jug r 1-sIgE levels increased after 1 month and decreased gradually until 12 months; walnut-sIgG4 levels increased

AE, adverse effects; CM, cow’s milk; CS, casein; EW, egg white; OFC, oral food challenge; OIT, oral immunotherapy; OM, ovomucoid; StU, short-term unresponsiveness; ω-5G, omega-5 gliadin.

### 4.1 Wheat

In 2019, a multicenter double blind randomized placebo-controlled trial was conducted (32). As many as 52.2% of participants in the low-dose OIT group, with a maintenance dose of 1,445 mg of wheat protein, achieved desensitization to 4,443 mg of wheat protein after one year. In contrast, none (0%) of the 23 placebo-treated subjects achieved desensitization (P < 0.0001). In 2020, Nagakura et al. conducted an open-label, non-randomized historically controlled trial (34), in which participants consumed 53 mg of wheat protein daily as a maintenance dose in the OIT group. Of these, 25% in the OIT group and 0% in the placebo group successfully passed the Oral Food Challenge (OFC) with 400 mg of wheat protein after one year (P = 0.07). In another OIT trial conducted in 2020 (6), 16.7% of subjects in the low-dose group, with a maintenance dose of 650 mg of wheat protein, achieved short-term unresponsiveness to 2,600 mg of wheat protein. The percentage of participants achieving short-term unresponsiveness in the higher maintenance dose group, with 2,600 mg of wheat protein, reached 50% after one year. Additionally, an open-label non-randomized controlled trial conducted by Shiro Sugiura et al. (33) found that in the slow low-dose OIT group, the proportion of participants who successfully achieved desensitization to 226 mg of wheat protein was slightly higher than that of the control group, although the difference was not statistically significant (37.5% vs. 10%, p=0.13). The incidence of adverse events varied significantly across all trials, but it was shown that severe adverse reactions were rare in low-dose OIT (6, 32, 33). Furthermore, it was observed that wheat- and omega-5 gliadin (ω-5G)-specific IgE (sIgE) levels decreased dramatically, while wheat-and ω-5G-specific IgG and IgG4 levels significantly increased in some trials (6, 33, 34).

### 4.2 Cow’s milk

In recent years, several trials on low-dose OIT for milk allergies have been conducted, as outlined in **Table 1**. It is evident that the proportion of participants who successfully achieved desensitization in the low-dose OIT group was higher than that in the placebo group (33, 35). However, there was no significant difference in the percentage of patients who were unresponsive to the target dose between the low-dose and high-dose OIT groups, despite a slightly higher percentage in the high-dose OIT group (6). Notably, the incidence of adverse reactions was significantly lower in the low-dose group compared to the high-dose group (6). Consistent with changes in allergen-specific IgE (sIgE), there was a significant decrease in milk-sIgE and casein-sIgE in both the high-dose and low-dose OIT groups. However, the studies did not specify whether there was any difference in allergen sIgE changes between the two groups (6).

### 4.3 Hens’ eggs

In line with the findings from low-dose OIT in wheat and milk allergy treatments, the proportions of desensitization among participants in high-dose, low-dose, and control groups in hens’ eggs OIT trials were largely consistent with those outlined in **Table 1**. Furthermore, the incidence of adverse reactions in low-dose OIT was relatively low, with no severe allergic reactions reported (36). Additionally, all studies indicated a significant decrease in egg white-specific IgE (EW-sIgE) and ovomucoid-specific IgE (OM-sIgE) (6, 33, 36). In the trial conducted by Noriyuki Yanagida et al. (36), it was demonstrated that egg white-specific IgG (EW-sIgG), ovomucoid-specific IgG (OM-sIgG), egg white-specific IgG4 (EG-sIgG4), and ovomucoid-specific IgG4 (OM-sIgG4) significantly increased after 12 months, with no significant difference observed in the placebo group.

### 4.4 Peanuts

Both trials conducted by Kenichi Nagakura et al. (70) and Katharina Blumchen et al. (31) demonstrated that after low-dose OIT for peanut allergies, the proportion of participants unresponsive to the target dose during OFC was significantly higher in the low-dose OIT group compared to the control group. Meanwhile, in a single-center, case-control randomized and double-blinded study by Brian P. Vickery et al. (5), participants were divided into a low-dose group (with a maintenance dose of 300 mg peanut protein) and a high-dose group (with a maintenance dose of 3,000 mg peanut protein). There was no significant difference in the proportion of participants unresponsive to 5,000 mg peanut protein after 29 months of OIT. However, adverse events (AEs) occurred more frequently in the high-dose OIT group compared to the low-dose group, although no severe allergic reactions were reported. During low-dose OIT, peanut and Arah2-specific IgE levels typically decreased dramatically, while peanut-specific IgG4 significantly increased. Furthermore, in a multi-center, randomized, and placebo-controlled trial (31), IL-4, IL-5, IL-10, and IL-2 production by PBMCs significantly decreased in the low-dose OIT group compared to the placebo-OIT group.

## 5 Low-dose OIT with hypoallergenic foods

For most foods, the heating process can induce conformational changes in allergen epitopes, reducing the reactogenicity of food allergens with IgE (37). Additionally, heat-denatured food allergens may stimulate Th1 polarization and the production of neutralizing IgG antibodies (72). Foods such as baked milk, heated milk, baked eggs, and hard-boiled eggs are occasionally used in food OIT (38, 41, 73). Currently, to minimize adverse events during OIT, particularly for patients with severe food allergies, many low-dose OIT trials have been conducted with hypoallergenic foods. We summarize studies on low-dose OIT with hypoallergenic foods in **Table 2**.

**Table 2:** Studies on low dose oral immunotherapy with hypoallergenic foods.

Food allergen	Reference	Design	Sample size and age	Patient characteristics	Maintenance dose	OIT dura-tion	Efficacy outcome	Safety outcome	Immunological parameter
**Milk**	Michael R et al. (2015/12) (73)	Open label non- randomized controlled trial	15 patients Age:78-145 months	Reacted initially to ≤30 mg of unheated CM protein	1.3g baked CM protein	12 months	Only three subjects achieved maintenance dose	Eight subjects dropped out because of IgE-mediated reactions.	CM-sIgE significantly decreased in the three subjects who achieved maintenance dose.
	Vianney Gruzelle et al. (2020/5) (38)	Single center, retrospective chart analysis	64 children, age: 4.8 (2-16) years	Had a recent (< 6 months) history of a type I allergic reaction for CM, CM sIgE>10 kUA/l, or casein sIgE>5 KUA/l	About 5ml baked milk	2 years	42.2% of children achieved desensitization to 8.6 g fresh CM protein	33.3% of the children had AEs during OIT, 18% OIT interruptions, no severe reaction.	CM sIgE and casein sIgE significantly decreased.
	Ken-ichi Nagakura et al. (2021/1) (37)	Randomized controlled trial	Unheated milk OIT: 6.1 (5.3-10.8) years; heated milk OIT: 7.6 (5.2-11.2) years.	With history of CM anaphylaxis; positive OFC with 3 mL heated milk.	Unheated milk OIT: 3 mL unheated milk. heated milk OIT: 3 mL heated milk	12 months	35% and 18% in the heated milk group and 50% and 31% in unheated milk group passed the 3 mL and 25 mL heated milk OFCs.	Any AE per dose: heated milk group 8.1%, unheated milk group 9.6%; P=0.01	Casein-sIgG4 significantly increased in both groups; a significant increase of β-lactoglobulin-sIgG4 in the unheated milk group.
**Egg**	Akihiro Maeta et al. (2018/1) (41)	Single center open label, non-placebo control study	Low dose OIT: n=13, 3 years 10 months to 8 years 7 months	13 children with egg allergy, recommended by their treating doctors, and they could not receive OIT using hard-boiled EW	79–110 mg of baked egg white protein	3-4 months	StU to 3.8 g of hard-boiled EW; Allergic severity was reduced in seven patients	It is safe without specifying the incidence.	OVA-sIgA2 levels, the ratios of OM-sIgG4/OM-sIgE and OM-sIgA2/OM-sIgE after OIT were significantly higher than before OIT.
	Yuri Takaoka et al. (2019/10) (74)	Single center double-blind, placebo-controlled study	Low dose OIT, n=19, 6 (2-9) years; placebo group, n=12 6 (1-9) years.	Positive OFC with 3.7 g hard-boiled egg white	Low dose: 79–110 mg of baked egg white protein control group: non-egg cookies	4 months	Compared with placebo group, in the low dose group, total OFC Aichi score for anaphylaxis/cumulative protein dose was lower and the proportion of StU was higher.	Any AE per dose: 5.1%. No severe symptoms occurred.	There was no significant difference in egg- and OM-sIgE and wheal diameter from the prick tests after 4 months between the two groups.
	Kim et al. (2020/1) (66)	Open-label randomized trial	Baked egg group (BE-R): n = 27, 7.2 ± 3.0 years; egg OIT group (OIT-R) n = 23, 9.1 ± 3.1 years; comparison group (OIT-A): n = 39, 8.8 ± 2.4 years	BE-R and OIT-R: negative DBPCFC with baked egg and positive with unbaked egg: DBPCFC with ≤ 1,444 mg of unbaked EW protein OIT-A. positive with baked egg	280-2,000 mg EW protein; BE-R. baked EW protein OIT-R and OIT-A: unbaked EW protein	2 years	StU to 7,444 mg of EW protein BE-R: 11.1% OIT-R: 43.5% (P = 0.009)	Any AE per dose: BE-R: 2.8% OIT-R: 3.9% OIT-A: 12.6% (BE-R vs OIT-R: P = 0.72; BE-R vs OIT-A: P = 0.008; OIT-R vs OIT-A: P=0.0138)	There was a significant decrease in EW-sIgE from baseline in all groups; there was a larger rise in EW-sIgG4 and egg component IgG4 in the OIT-R and OIT-A groups than that in the BE-R group.
	Yuri Takaoka et al. (2023/4) (42)	Single-center non-inferiority randomized, low- and high-dose trial of open-labeled	Low dose OIT group, n=23, 3-12 years; high dose OIT group, n=29, 3-15 years	Positive OFC with 38 g boiled EW	Hard-boiled EW; low dose OIT 2g; high dose OIT, 20g	6 months	There were three patients in each group who tested negative in OFC with a 20 g hard-boiled EG. (p = 1.000)	Any AE per dose: low dose OIT: 5.6%; high dose OIT: 5.7%.	EW-sIgE and OM-sIgE showed a significant decrease after 6 months in both groups.

AE, adverse effect; CM, cow’s milk; CS, casein; DBPCFC, double-blind placebo- controlled food challenge; EW, egg white; OFC, oral food challenge; OIT, oral immunotherapy; OM, ovomucoid; StU, short-term unresponsiveness.

### 5.1 Cow’s milk

In children receiving low-dose OIT for milk allergies, with a maintenance dose of 5ml baked milk, milk-specific IgE (sIgE) and casein-specific IgE (sIgE) significantly decreased (38). This resulted in 42.2% of children achieving desensitization to 254ml fresh milk. Moreover, in a randomized controlled trial with unheated milk and heated milk by Ken-ichi Nagakura et al. (37), casein-specific IgG4 significantly increased in both groups from baseline. However, a significant increase of β-lactoglobulin-specific IgG4 was only detected in the unheated milk group. In the unheated milk group, the proportion of children who successfully passed the OFC was significantly higher than that in the heated milk group. As expected, more adverse events occurred in the unheated milk group compared to the heated milk group, suggesting that while low-dose OIT with hypoallergenic foods may improve safety, it may also reduce efficacy.

### 5.2 Hens’ eggs

Several clinical trials on low-dose OIT with hypoallergenic egg products have been conducted. Among these, a single-center open-label, non-placebo-controlled study with a maintenance dose of 79–110 mg of baked egg white (EW) protein conducted by Akihiro Maeta et al. (41) demonstrated that ovalbumin-specific IgA2 (OVA-sIgA2) levels after OIT were significantly higher than baseline. Additionally, the ratios of ovomucoid-specific IgG4 to IgE (OM-sIgG4/OM-sIgE) and ovomucoid-specific IgA2 to IgE (OM-sIgA2/OM-sIgE) after OIT were also significantly higher compared to those before OIT. Among participants who were severely allergic to eggs and could not tolerate hard-boiled EW, more than 50% achieved unresponsiveness to 3,800 mg of hard-boiled EW.

In a single-center, double-blind, and placebo-controlled study (74), where 79–110 mg of baked EW protein was set as the maintenance dose, the proportion of patients reaching unresponsiveness in the low-dose group was higher than that in the placebo group after a 4-month OIT. However, there was no significant difference in egg- and ovomucoid-specific IgE (OM-sIgE), or even in the wheal diameter from the prick tests between the two groups. In an open-label randomized trial (66) for children who were negative with a double-blind placebo-controlled food challenge with baked egg and positive with 1,444 mg unbaked egg, children were randomly divided into two low-dose OIT groups with baked EW protein and unbaked EW protein, respectively. The proportion of children reaching unresponsiveness to 7,444 mg EW protein in the unbaked EW protein group was higher than that in the baked EW protein group after a 2-year OIT. This suggests that low-dose OIT with common food is more conducive to inducing tolerance compared to low-dose OIT with hypoallergenic foods, possibly due to the larger increase of IgG in the unbaked EW protein group compared to the baked EW protein group. However, there was a significant decrease in EW-specific IgE (EW-sIgE) from baseline in both groups. In a recent study (42) using 2,000 mg and 20,000 mg of hard-boiled EW as the maintenance dose in the low-dose group and high-dose group, respectively, not only the desensitization effect but also EW-sIgE and ovomucoid-specific IgE (OM-sIgE) showed no significant difference. Notably, as **Table 3** shows, some conventional dose OIT with baked egg and dehydrated egg were carried out. They all significantly promoted tolerance formation without any severe allergic reaction (39, 51, 75). EW-sIgE and OM-sIgE significantly decreased, and specific IgG4 increased, while both CD203c^+^ and CD63^+^ basophils saw a more significant decrease over time in the OIT group than that in the placebo group (51).

**Table 3:** Studies on conventional dose oral immunotherapy with hypoallergenic foods.

Food allergen	Reference	Design	Sample size and age	Patient characteristics	Maintena-nce dose	OIT duration	Efficacy outcome	Safety outcome	Immunological parameter
**Egg**	Garcia et al. (2012) (75)	Open prospective study	16 children mean age: 7.88 years; three adults mean age: 28.3 years	History suggestive of immediate type allergic reaction after ingestion of egg	3.6 g dehydrated egg white powder	13 months	14 patients reached the maintenance dose and tolerated at least 3 HEs per week	70.5% subjects developed symptoms; these were mild or moderate in most cases.	Not mentioned
	Vianney Gruzelle et al. (2021/7) (39)	Single center, retrospective analysis	71 children, age: 6 years (2-17) years	Had type I allergic reaction to egg, with high sIgE: >2years, EW sIgE > 7 kUA/L <2years, >2 kUA/L	5 g baked egg	1.3 years	Desensitization to 5 g of unbaked egg protein: 66.2%.	No anaphylactic reaction to baked egg OIT.	EW sIgE and OM -sIgE significantly decreased.
	Giavi et al. (2016/11) (51)	Pilot multicenter study; a double-blind placebo-controlled pilot study	1-5.5 years; OIT: 15; control: 14	A positive SPT to egg white, positive OFC, or immediate allergic reaction to egg	OIT: 9 g dehydrated egg white powder; control: placebo	6 months	OFC was passed; OIT group: 36%; placebo group: 21% (p>0.05)	OIT group: 7 AEs; placebo group: two AEs. No severe events.	EW-sIgG4 increased; CD203c^+^ and CD63^+^ basophils decreased more in the OIT group than that in the placebo group.

AE, adverse events; EW, egg white; HE, hen’s egg; OFC, oral food challenge; OIT, oral immunotherapy; OM, ovomucoid.

## 6 Conclusions

The mechanisms underlying low-dose OIT share some similarities with those of conventional OIT, although they have not been fully elucidated and require further exploration. Low-dose OIT, particularly when applied with hypoallergenic foods, may induce tolerance with significantly fewer adverse events compared to conventional OIT. However, while safer, low-dose OIT and low-dose OIT with hypoallergenic foods may not be as effective as conventional OIT in inducing tolerance. The results can be influenced by numerous factors, such as the sample size and the severity of allergies in participants. Therefore, further research is needed on low-dose OIT, ideally through large-sample, multicenter, and double-blind studies.

## Author contributions

DM: Funding acquisition, Writing – original draft, Writing – review & editing. RZ: Conceptualization, Supervision, Writing – review & editing.
